# The Extracts of *Angelica sinensis* and *Cinnamomum cassia* from Oriental Medicinal Foods Regulate Inflammatory and Autophagic Pathways against Neural Injury after Ischemic Stroke

**DOI:** 10.1155/2021/9663208

**Published:** 2021-06-26

**Authors:** Cheng Luo, Qi Chen, Bowen Liu, Shengpeng Wang, Hualin Yu, Xiaowei Guan, Yonghua Zhao, Yitao Wang

**Affiliations:** ^1^The Second Department of Neurosurgery, First Affiliated Hospital of Kunming Medical University, Kunming, China; ^2^The Affiliated Hospital of Southwest Medical University, Luzhou, China; ^3^State Key Laboratory of Quality Research in Chinese Medicine, Institute of Chinese Medical Sciences, University of Macau, Macao, China; ^4^Department of Human Anatomy and Histoembryology, School of Medicine and Life Sciences, Nanjing University of Chinese Medicine, Nanjing, China

## Abstract

The study indicates inflammation and autophagy are closely related to neural apoptosis in the pathology of ischemic stroke. In the study, we investigate the effects and mechanisms of the extracts of *Angelica sinensis* and *Cinnamomum cassia* (AC) from oriental medicinal foods on inflammatory and autophagic pathways in rat permanent middle cerebral artery occlusion model. Three doses of AC extract were, respectively, administered for 7 days. It suggests that AC extract treatment ameliorated scores of motor and sensory functions and ratio of glucose utilization in thalamic lesions in a dose-dependent manner. Expression of Iba1 was decreased and CD206 was increased by immunofluorescence staining, western blotting results showed expressions of TLR4, phosphorylated-IKK*β* and I*κ*B*α*, nuclear P65, NLRP3, ASC, and Caspase-1 were downregulated, and Beclin 1 and LC3 II were upregulated. Low concentrations of TNF-*α*, IL-1*β*, and IL-6 were presented by ELISA assay. Additionally, caspase 8 and cleaved caspase-3 expressions and the number of TUNEL positive cells in ipsilateral hemisphere were decreased, while the ratio of Bcl-2/Bax was increased. Simultaneously, in LPS-induced BV2 cells, it showed nuclear P65 translocation and secretion of proinflammatory cytokines were suppressed by AC extract-contained cerebrospinal fluid, and its intervened effects were similar to TLR4 siRNA treatment. Our study demonstrates that AC extract treatment attenuates inflammatory response and elevates autophagy against neural apoptosis, which contributes to the improvement of neurological function poststroke. Therefore, AC extract may be a novel neuroprotective agent by regulation of inflammatory and autophagic pathways for ischemic stroke treatment.

## 1. Introduction

Stroke has been a top-ranked cause of mortality and disability in both the 50–74-year and 75-years-and-older age groups, globally [1]. Prevalent data of stroke show that 84.4% of the total number belongs to ischemic stroke [2]. Numerous literatures have indicated that neuroinflammatory response plays an essential role in the propagation of brain damage after stroke [3]. Due to cerebral vascular occlusion by thrombosis at the onset of ischemic stroke, regional cerebral blood flow (CBF) instantaneously decreases and stagnates, which induces shear stress on endothelial cells (ECs) and platelets resulting in excessive adhesion molecules secretion, contributing to circulating leukocytes recruitment and binding onto ECs. It exacerbates coagulation cascades and initiates the earliest inflammatory response [4]. Following the disruption of blood-brain barrier (BBB), circulating leukocytes are apt to infiltrate into brain parenchyma. Leukocyte infiltration combined with danger-/damage-associated molecular patterns (DAMPs) from injured and dying neurons triggers a cerebral inflammatory cascade as a marker of activation of microglia and astrocytes [5]. DAMPs activate Toll-like receptors (TLRs) of subfamilies of pattern recognition receptors (PRRs) on microglia, astrocytes, and oligodendrocytes and activated TLRs, like TLR4, interacting with the myeloid differentiation primary-response protein 88 (MyD88) upregulates nuclear factor-kappa B (NF-*κ*B) signaling [6, 7]. As another subfamilies of PRRs, Nod-Like receptors (NLRs) located in the cytoplasm primarily involved in the formation of NLR proteins (NLRPs) inflammasome which exerts important action in the neurovascular unit and has been a novel target on vascular diseases [8]. NLRP3 inflammasome, as the most featured member of the NLR family, participates in neuronal apoptosis after ischemic stroke [9]. Presently, NF-*κ*B signaling has been demonstrated to involve in the activations of NLRP1 and NLRP3 inflammasomes, and inhibition of the pathway not only decreases interleukin- (IL-) 1*β*, IL-18, tumor necrosis factor- (TNF-) *α*, and IL-6 but also attenuates apoptotic cells in cerebral tissue after cerebral ischemia [10, 11]. Therefore, the inhibition of neuroinflammatory pathways is a crucial target for cerebral protection poststroke.

Recent research reports that moderate autophagy rescues injured cells by removing damaged tissues and proteins [12, 13]. Autophagy has closed interaction with apoptosis by multiple mechanistic overlaps. Evidence illustrates that mitochondria-dependent intrinsic apoptotic pathway activating caspase-3 not only arouses apoptotic cell death but also inhibits autophagy in ischemic stroke resulting from antiapoptotic protein Bcl-2 binding to Beclin-1 [13], so suppressed apoptosis and increased autophagy contributed to the recovery of hippocampus injury in cerebral ischemia/reperfusion rats [14]. Although mechanisms between autophagy and inflammation are not clear at present, it indicates that autophagy negatively regulates inflammasome activation against inflammation, whose efficacies mainly embody that the phagocytic actions of autophagy advance apoptotic corpse clearance as well as autophagy transports inflammasome components into lysosomes for degradation and sequesters IL-1*β* [15, 16]. It is necessary to develop innovative pharmacological agents targeting inflammatory and autophagic pathways in ischemic stroke.

Chinese herbs begin to systematically treat stroke before 1800 years and have accumulated abundant clinical experiences and effective prescriptions. Emerging studies suggest that compounds extracted from Chinese herbs exert significant neuroprotective effects via multiple approaches [17]. *Angelica sinensis* (Oliv.) Diels, root and rhizome, and *Cinnamomum cassia* (L.) J. Presl, stem bark, as oriental herbs from medicinal foods, their combination had been reported to be used to treat stroke in Chinese medicinal literature before 300 years [18]. They are widely used as either traditional medicines or functional foods in East and South-East Asia countries such as Korea, Japan, India, Thailand, Vietnam, and Indonesia [19, 20]. In the study, we investigate neuroprotective effects of the extracts of *Angelica sinensis* and *Cinnamomum cassia* (AC) on cerebral tissue and hypothesize the mechanisms might be related to the regulation of inflammatory and autophagic pathways following cerebral ischemia.

## 2. Materials and Methods

### 2.1. The Extracted Method and Quality Control

The root and rhizome of *Angelica sinensis* (Oliv.) Diels and stem bark of *Cinnamomum cassia* (L.) J. Presl, were purchased from Guangzhou Zisun Pharmaceutical Co., Ltd., China, which are accordant with the standards of Chinese Pharmacopoeia (2015 edition) by confirmation of Professor Quan Zhu. We performed the method of steam distillation for the extracts of AC (1 : 1 of two herbal medicine weight ratio), and detailed procedures refer to our previous article [21]. The extracts (yield: 25%) were obtained by lyophilizing the concentrated sample with a Virtis Freeze Dryer (The Virtis Company, New York, USA). 5 mg of AC extract was dissolved in 5 ml of 30% methanol and filtered, which was directly subjected to ACQUITY UPLC system on PAD *λ*e detector (*λ* = 200–400 nm), autosampler, in-line degasser, Waters ACQUITY-UPLC CLASS system (Waters Corp., Milford, USA) on an ACQUITY UPLC HSS T3 column (150 mm × 2.1 mm, 1.8 *μ*m). Reference compounds are ferulic acid (*t* *R* = 2.59 min, purity HPLC > 98%), Senkyunolide I (*t* *R* = 4.42 min, purity HPLC > 98%), and Trans-Cinnamic acid (*t* *R* = 6.32 min, purity HPLC > 98%), which were purchased from Chengdu Chroma-Biotechnology Co., Ltd., China.

### 2.2. Middle Cerebral Artery Occlusion Model and Group Division

Male Sprague-Dawley (SD) rats weighing 220-250 g were provided by Kunming Medical University Laboratory Animal Services Center. All animal operations conformed to the NIH Guide for the Care and Use of Laboratory Animals (National Institutes of Health Publication No. 85–23, revised in 1985), and University of Macau ethical committee approved the experimental protocol. Permanent middle cerebral artery occlusion (MCAo) model was established according to our previous report [21]. Briefly, 2% pentobarbital sodium (4 mL/kg) was used to anesthetize SD rats by intraperitoneal injection, and subsequently, middle cerebral artery was occluded by inserting 4-0 surgical nylon suture coated with polylysine. Simultaneously, rats in the sham group were subject to the same administered procedure except for suture occlusion. All operated rats were put on animal heating pads to maintain their rectal temperature at 37°C, and only MCAo rats with a score of 2 assessed by 5-point scale neurological deficit score [22] were randomly divided into four groups after they recovered consciousness.

Treatment groups include low dosage of AC extract (Low) group, middle dosage of AC extract (Middle) group, and high dosage of AC extract (High) group, and 1.6 g/kg, 3.2 g/kg, and 6.4 g/kg of AC extract solved in distilled water were, respectively, administered to rats by gavage once daily for 3 days before operation and continuing to 7 days after MCAo surgery. Rats in MCAo and Sham groups were given the same volume of distilled water.

### 2.3. The Harvest of Cerebrospinal Fluid

At day 7, rats (*n* = 6) in sham, MCAo, and high dose of AC extract groups were intraperitoneally injected into 2% pentobarbital sodium (4 mL/kg) for deep anesthetization, and then 1 mL injector was slowly inserted into posterior atlantooccipital membrane avoiding arteria spinalis dorsalis to harvest 30 *μ*L CSF from each rat. Collections of CSF in each group were, respectively, termed sham group's, MCAo group's, and high dose group's CSF.

### 2.4. MicroPET/CT Imaging and Neurological Functional Assessment

^18^F-FDG-PET/CT imaging was performed to evaluate glucose uptake of rats (*n* = 5) in ipsilateral hemisphere at days 1, 3, and 7. The detailed performance procedure was described in our previous article [23]. Briefly, rats in each group were anesthetized with isoflurane (3.0% in air) asphyxiation, then, intravenously administered with 37 MBq (∼1 mCi) of ^18^F-FDG. After 60 min later, microPET/CT images and CT images were, respectively, acquired for 30 min using a FLEX X-PET and X-O small animal imaging system (TriFoil) and with 256 projections over 2 min for attenuation correction and anatomy landmarks. Commercial software (Visage Imaging) with 72 *μ*m isotropic CT spatial resolution and 2 mm for PET imaging was used for the coregistered images of PET and CT. For quantitative analysis, volume of interests (VOIs) were, respectively, drawn on six regions (Amygdala, Caudate Putamen, Cortex Motor, Cortex Somatosensory, Hypothalamus and Thalamus Whole) of contralateral and ipsilateral hemispheres. Percent-injected dose of ^18^F-FDG per c.c. of brain tissue (%ID/cm^3^) was obtained from each VOI. Metabolic ratio of each region was calculated with the following formula: ratio = %ID/c.c.of lesion region/%ID/c.c.of contralateral side.

Additionally, we invited a colleague who was unknown to the experimental group division to evaluate the amelioration of neurological function in rats poststroke. At day 7 after ischemia, assessment of motor recovery (*n* = 6) including spontaneous activity, symmetry in the movement of four limbs, forepaw outstretching, and climbing, and test of sensory recovery (*n* = 6) including body proprioception and response to vibrissae touch [24] were performed.

### 2.5. TLR4 siRNA Transfection in BV2 Microglial Cells

BV2 microglial cell lines purchased from China Center for Type Culture Collection were seeded at a density of 2.5 × 10^5^ cells/ml into 6-well dishes and cultured with Dulbecco's modified Eagle's medium (Gibco; Thermo Fisher Scientific, Inc., USA) supplemented with 10% fetal bovine serum at 37°C with 5% CO_2_. Cells were transfected with siRNA against TLR4, when they presented logarithmic growth to 70% confluent. According to manufacturer's protocol, BV2 microglial cells were transfected with TLR4 siRNA consisting of three target-specific 19-25 nt siRNAs to knock down gene expression using siRNA Transfection Reagent (CAT#: sc-40261, Santa Cruz Biotechnology, Inc.). Negative control of TLR4 siRNA transfection contained a scrambled sequence that did not cause specific degradation of any known cellular mRNA (CAT#: sc-37007, Santa Cruz Biotechnology, Inc.).

### 2.6. Lipopolysaccharide-Stimulated BV2 Microglial Cells Incubated with CSF

Before lipopolysaccharide- (LPS-) stimulated BV2, 15% high dose group's CSF was, respectively, added into BV2 and TLR4 siRNA-transfected BV2. Simultaneously, 15% MCAo group's CSF was, respectively, added into BV2, TLR4 siRNA, and negative TLR4 siRNA-transfected BV2. 15% Sham group's CSF added into BV2 was used to be as normal control. Subsequently, 100 ng/mL of LPS derived from *Escherichia coli* O111:B4 (Sigma-Aldrich, Inc. Cat#: L4391) was employed to stimulate these BV2 cells for 24 hours based on preliminary experiments and other reports [25], except BV2 in the normal group. Ultimately, LPS+ high dose group's CSF (High), LPS + TLR4 siRNA+High, LPS, LPS + TLR4 siRNA, and LPS + negative TLR4 siRNA groups were formed.

### 2.7. Western Blotting

At day 7, rats (*n* = 3) in five groups were sacrificed by excessive anesthesia and transcardially perfused with 4°C sterile saline. Total proteins were extracted from the ipsilateral hemisphere of each group as well as different processed BV2 in vitro, and nuclear/cytoplasmic proteins were isolated using NE-PER Nuclear and Cytoplasmic Extraction Kit (Thermo Scientific™ 78833, USA). Protein concentration was determined by a bicinchoninic acid protein assay kit (Beyotime Institute of Biotechnology, Shanghai, China). Equal amounts of proteins were separated on 10% sulfate-polyacrylamide gel electrophoresis and transferred to polyvinylidene fluoride membranes. Subsequently, membranes were blocked in 5% (*w*/*v*) skimmed dried milk for 1 h at room temperature and incubated with primary antibodies for anti-TLR4 (1 : 2000, Signalway Antibody), anti-IKK*β* (1 : 2000, CST), anti-p-IKK*β* (1 : 1500, CST), anti-I*κ*B*α* (1 : 1500, CST), anti-p-I*κ*B*α* (1 : 1500, CST), anti-P65 (1 : 2000, CST), anti-NLRP3(1 : 2000, Signalway Antibody), anti-ASC (1 : 2000, Signalway Antibody), anti-Caspase1(1 : 2000, CST), anti-cleaved Caspase 3,8 (1 : 2000, CST), anti-Bcl-2 (1 : 2000, CST), anti-Bax (1 : 2000, CST), anti-Beclin 1 (1 : 2000, SAB), anti-LC3 (1 : 2000, CST), *β*-actin (1 *μ*g/mL, Abcam), and Lamin B1 (1 : 1000, sc-374015, Santa Cruz Biotechnology, Inc., USA) at 4°C overnight. The membranes were gently washed with TBST [50 mM Tris-HCl (pH 7.4; Acros Organics BVBA, Geel, Belgium), 150 mM NaCl, 0.05% Tween 20 (Acros Organics BVBA)] three times and incubated with goat anti-rabbit IgG (H &L) or goat anti-mouse IgG (H &L) secondary antibodies (1 : 5000; Thermo Fisher Scientific, USA) at room temperature for 1 h. Signals of reactive bands were visualized by fluorescence scanner (LI-COR, Lincoln, Nebraska, USA), and quantitative analysis of targeted proteins was by ratio of corresponding proteins to *β*-actin or Lamin B1 (nuclear internal control), and phosphorylated levels of IKK*β* and I*κ*B*α* were analyzed by total levels of corresponding proteins. Western blots were duplicated three independent times.

### 2.8. TUNEL Assay

Rats (*n* = 3) were sacrificed at day 7, and their brain tissues were fixed with fresh 4% paraformaldehyde solution. Fresh frozen coronal sections of 8 *μ*m thickness were cut by a cryostat microtome (Shandon Cryotome FSE, Thermo Fisher Scientific, USA). Subsequently, slices were fixed again for half an hour in 4% paraformaldehyde. After washing with PBS for three times, slices were incubated with 0.3% Triton X-100 at 4°C for 15 min. TUNEL assay (MA0224, MeiLunBio®, CHA) was performed according to the manufacturer's recommendations.

### 2.9. Immunofluorescence Staining

Every fifth slice of a total section was used to detect activations of microglia in ipsilateral hemisphere by incubation with primary antibody against Iba1 (1 : 200, Abcam, UK) and anti-CD206 (1 : 500, Abcam, UK) at 4°C for overnight. BV2 cells in six groups seeded on slides were fixed with 4% paraformaldehyde for 30 min at 4°C, permeabilized with 0.3% Triton X-100 for 15 min, and incubated with anti-P65 (1 : 500, CST) overnight. Both brain tissue and cell slides were washed with PBS and, respectively, incubated with the bs-0294D-FITC (1 : 200, Beijing Biosynthesis Biotechnology Co., Ltd., CHA) and Alexa Fluor® 488 goat anti-rabbit immunoglobulin (1 : 200, Life Technologies) secondary antibodies for 1 h at room temperature. Nuclei were stained with 4′, 6-diamidino-2-phenylindole (DAPI; Sigma). The number of BV2 with positive signal of nuclear P65 was calculated in five randomly selected microscopic fields at 400× magnification. Fluorescent labeling of Iba1, CD206, and TUNEL were observed with the Leica TCS SP8 laser scanning confocal microscope (Leica Microsystems Inc., Buffalo Grove, USA), and five nonoverlapping fields of one slice in the penumbral cortex were measured. Image-Pro Plus software (Media Cybernetics, Rockville, MD, USA) was used for quantitative analysis.

### 2.10. ELISA Assay

TNF-*α*, IL-1*β*, and IL-6 in serum of peripheral blood, CSF, ipsilateral hemisphere, and conditional mediums from BV2 were measured using ELISA assay. At day 7, 5 ml blood was extracted from the abdominal aorta in deeply anesthetized rats (*n* = 3) after 2 hours of the last gavage and placed it in tubes at room temperature for 1 ~ 2 hours before it became condensation at 4°C. Finally, blood sample was centrifuged at 4°C 2000 rpm for 20 min to separate the serum. Ipsilateral hemisphere was homogenized in PBS using a tissue homogenizer, followed by incubated with the addition of 200 *μ*L of radioimmunoprecipitation assay buffer on ice for 10 min. Then, samples were centrifuged at 12500 × g for 5 min at 4°C, and supernatant was obtained. Conditional mediums were collected at time point after BV2 incubated with different CSF for 24 hours. TNF-*α* (Cat No: RTA00), IL-1*β* (Cat No: RLB00), and IL-6 (Cat No: R6000B) ELISA kits (R&D Systems, USA) were performed according to manufacturer's instructions. Optical density (OD) was measured at a mean wavelength of 570 nm by using SpectraMax Paradigm Multi-Mode Microplate Reader (San Jose, CA, USA).

### 2.11. Statistical Analysis

Continuous variables were presented based on the average ± standard deviation, and GraphPad Prism 5.0 (GraphPad Software, Inc.) was applied to the statistical analysis. One-way ANOVA was used to compare two means from two independent groups followed by Tukey's multiple comparisons test. A value of *p* < 0.05 was considered statistical significance in all analyses.

## 3. Results

### 3.1. Quality Control Results of AC Extract

**Figure 1:**
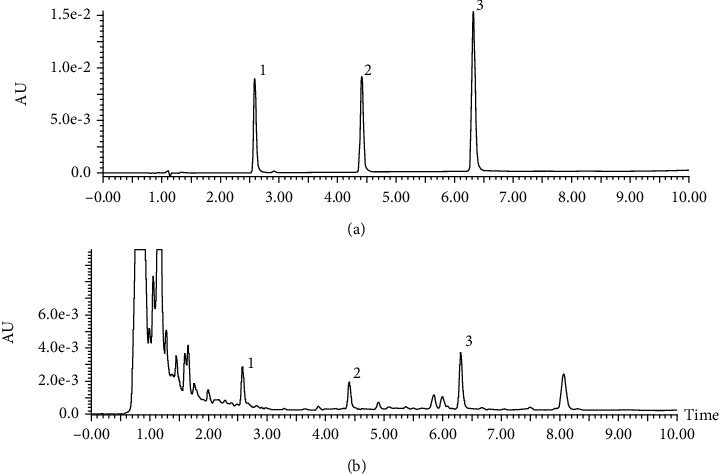
Quality control of AC extract by HPLC. Representative HPLC chromatogram of mixed standard solution (a) and AC extracts (b) with ferulic acid (1), Senkyunolide I (2), and Trans-Cinnamic acid (3); UV detection *λ* = 203 nm.

Quality control results by HPLC analysis indicated that the content of ferulic acid was 0.042 ± 0.001%, the content of Senkyunolide I was 0.021 ± 0.001%, the content of Trans-Cinnamic acid is 0.026% in AC extract, and the chromatographic peaks of ferulic acid, Senkyunolide I, and Trans-Cinnamic acid in AC extract, as well as the peak of reference solution were shown in Figure 1.

### 3.2. AC Extract Treatment Ameliorated Cerebral Glucose Utilization and Neurological Outcome

**Figure 2:**
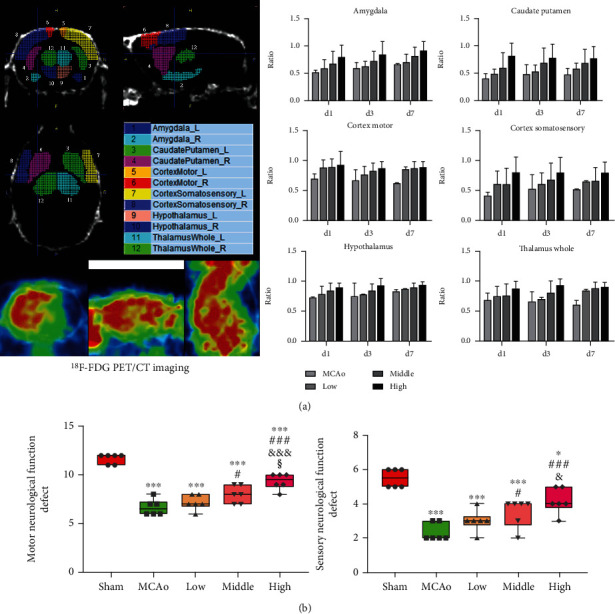
Cerebral glucose utilization and neurological function assessment. (a) Metabolic ratio of ^18^F-FDG in 6 regions of brain was presented. (b) Evaluation of motor and sensory function scores. Data presented as mean ± standard deviation. ^∗^*p* < 0.05, ^∗∗∗^*p* < 0.001 vs. Sham; ^#^*p* < 0.05, ^###^*p* < 0.001 vs. MCAo; ^&^*p* < 0.05, ^&&&^*p* < 0.001 vs. low; ^§^*p* < 0.05 vs. middle.

As shown in Figure 2(a), AC extract treatment improved glucose utilization in six ipsilateral lesion regions in a dose-dependent manner. Especially in Thalamus Whole region, the ratio in high dose group was obviously higher than that in MCAo group at day 3 (*p* < 0.05). Outcomes of neurobehaviors test at day 7 in Figure 2(b) suggested that motor function in high dose group was the most significant amelioration among three therapeutic groups (*p* < 0.05 vs. middle dose, *p* < 0.001 vs. low dose). Simultaneously, sensory functional outcome also suggested the same improved trend, and attenuated sensory deficiency in high dose group was more obvious than that in low dose group (*p* < 0.05).

### 3.3. AC Extract Treatment Suppressed Proinflammatory Microglial Phenotype

**Figure 3:**
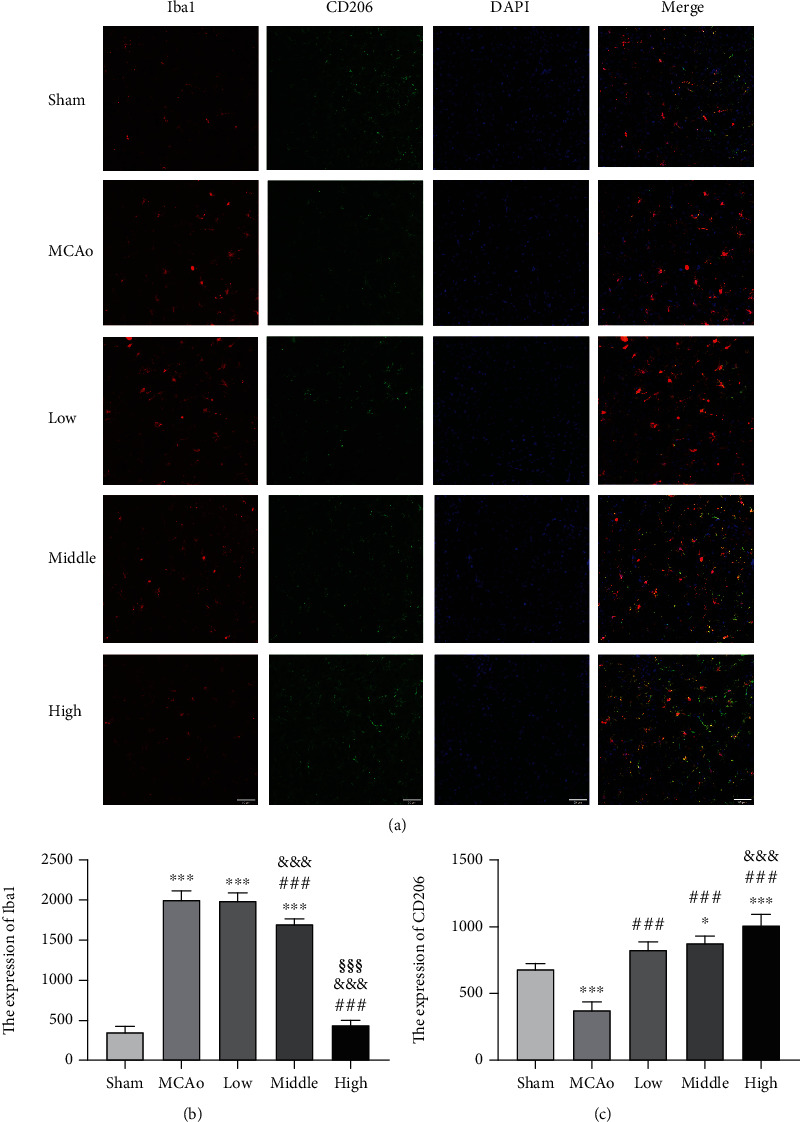
Microglial inflammatory phenotypes in ischemic boundary zone after stroke. (a) Immunofluorescence staining images of Iba1 (red) and CD206 (green) were presented (magnification, ×400). (b and c) Relative expressions of Iba1 and CD206 in ischemic cortex zone were analyzed. Scale bar 50 *μ*m. Data presented as mean ± standard deviation. ^∗∗∗^*p* < 0.001 vs. sham; ^###^*p* < 0.001 vs. MCAo; ^&&&^*p* < 0.001 vs. low; ^§§§^*p* < 0.001 vs. middle.

As classic biomarkers of microglia, increasing Iba1 expression represents microglial activation, and CD206 is regarded as an anti-inflammatory marker of microglia. As shown in Figure 3(a), the images of immunofluorescence staining showed that positive signals of Iba1 (red) and CD206 (green) widely distributed in ipsilateral hemisphere, and there existed sporadic overlapping signals (yellow). Quantitative analysis indicated that Iba1 expression in MCAo group was distinctly ascended (*p* < 0.001, vs. middle-high dose groups), while treatment with AC extract reversed the results in a dose-dependent manner at day 7 (Figure 3(b)). Simultaneously, expressions of CD206 in three dose groups suggested a significantly increasing trend compared with that in MCAo group (*p* < 0.001, Figure 3(c)). The results illustrated that AC extract treatment inhibited activated microglial cells and facilitated them to transform to anti-inflammatory phenotype after ischemic stroke.

### 3.4. AC Extract Treatment Inhibited TLR4/NF-*κ*B Pathway, NLRP3 Inflammasome Assembly, and TNF-*α*, IL-1*β*, and IL-6 Secretion

**Figure 4:**
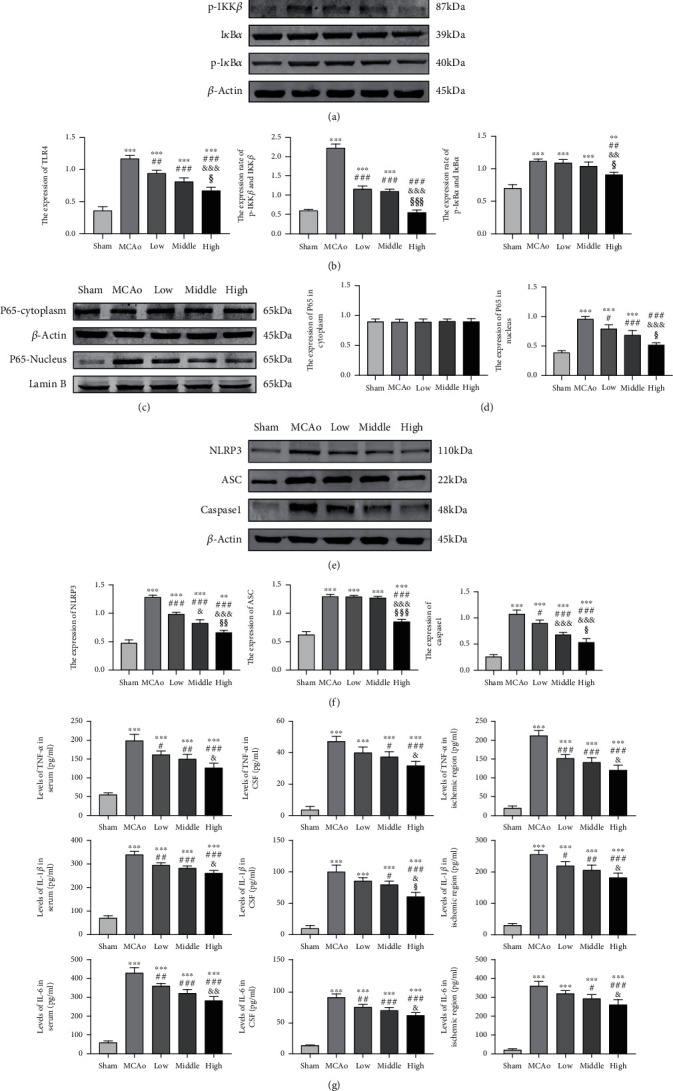
TLR4/NF-*κ*B pathway, NLRP3 inflammasome, and inflammatory cytokines poststroke. (a, c, and e) Representative western blot belts for TLR4, phosphorylated IKK*β* and I*κ*B*α*, nuclear and cytoplasmic P65, NLRP3, ASC, and caspase-1 in each group's ipsilateral hemisphere. (b, d, and f) Quantitative analysis of TLR4/NF-*κ*B pathway and NLRP3 inflammasome. (g) ELISA assay of TNF-*α*, IL-1*β*, and IL-6 in serum of peripheral blood, CSF, and ipsilateral hemisphere. Data presented as mean ± standard deviation. ^∗∗^*p* < 0.01, ^∗∗∗^*p* < 0.001 vs. sham; ^#^*p* < 0.05, ^##^*p* < 0.01, ^###^*p* < 0.001 vs. MCAo; ^&^*p* < 0.05, ^&&^*p* < 0.01, ^&&&^*p* < 0.001 vs. low; ^§^*p* < 0.05, ^§§§^*p* < 0.001 vs. middle.

Western blotting results showed that treatment with a high dose of AC extract not only notably suppressed TLR4 expression but also inhibited phosphorylation of IKK*β* and I*κ*B*α* compared with low-middle dose treatment (*p* < 0.05, Figures 4(a) and 4(b)). Nuclear P65 expressions in tree treatment groups indicated dose-dependent response to descend compared with that in MCAo group (*p* < 0.001, Figures 4(c) and 4(d)), and the expression of P65 in cytoplasm had no obvious alteration among five groups. As shown in Figure 4(e), expressions of NLRP3, ASC, and cleaved caspase-1 obviously elevated in MCAo group at day 7, suggesting stroke stress-activated NLRP3 inflammasome assembly. Quantitative analysis showed that AC extract treatment prevented assembly of NLRP3 inflammasome by reducing NLRP3, ASC, and caspase-1 expressions, and high dose of AC extract presented the most predominantly inhibited action among three doses groups (*p* < 0.05, vs. low-middle dose group, Figure 4(f)).

Quantitative analysis suggested that the amount of TNF-*α* in CSF and supernatant of ipsilateral hemispheric homogenizer was more than 8 ~ 10 folds in MCAo group as much as that in sham group, and its amount in serum of peripheral blood was 4 folds higher than that in the sham group. In the MCAo group, changed trends of IL-1*β* and IL-6 were parallel with that of TNF-*α* in CSF, brain parenchyma, and serum. The results showed that concentrations of TNF-*α*, IL-1*β*, and IL-6 in the brain were notably ascended compared with those in peripheral blood poststroke. High dose of AC extract group distinctly reduced TNF-*α*, IL-1*β*, and IL-6 concentrations whatever in CSF or brain parenchyma and serum of peripheral blood in comparison with low dose group (*p* < 0.05, Figure 4(g)).

### 3.5. AC Extract Treatment Decreased Nuclear P65 Translocation and TNF-*α*, IL-1*β*, and IL-6 Concentrations via Targeting TLR4 in LPS-Stimulated Microglial Cells

**Figure 5:**
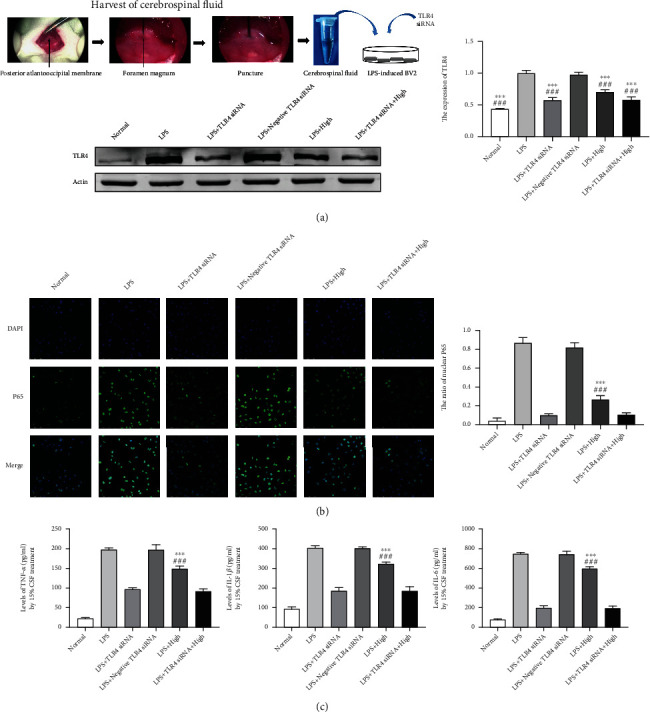
TLR4 and nuclear P65 expressions and inflammatory cytokines concentrations in LPS-stimulated BV2 microglial cells. (a) Schematic diagram of LPS-stimulated BV2 microglial cells incubated with CSF and transfected with TLR4 siRNA, as well as TLR4 expression by western blotting. (b) Immunofluorescence staining with nuclear p65 (green) was presented in LPS-induced BV2 microglial cells (magnification, ×400) and positive signal analysis. (c) ELISA assay of TNF-*α*, IL-1*β*, and IL-6 in conditional culture medium. Data presented as mean ± standard deviation. ^∗∗∗^*p* < 0.001 vs. LPS; ^###^*p* < 0.001 vs. LPS + negative TLR4 siRNA. Scale bar 50 *μ*m.

It indicated that TLR4 expression and the number of BV2 with nuclear P65 positive signal in LPS + High group were obviously decreased compared with those in LPS and LPS + negative TLR4 siRNA groups (*p* < 0.001, Figures 5(a) and 5(b)). Furthermore, combination treatment of TLR4 siRNA and high dose group's CSF did not further reduce the number of BV2 with nuclear P65 positive signal, showing AC extract should target TLR4 against nuclear P65 translocation in LPS-stimulated microglial cells.

In vitro, quantitative analysis in Figure 5(c) indicated concentrations of TNF-*α*, IL-1*β*, and IL-6 were high in LPS and LPS + negative TLR4 siRNA groups, but this inflammatory cytokine production was inhibited in LPS + High and LPS + TLR4 siRNA groups. Simultaneously, TLR4 siRNA plus high dose group's CSF treatment failed to further downregulate concentrations of TNF-*α*, IL-1*β*, and IL-6, suggesting AC extract treatment decreased inflammatory cytokines secretion via antagonizing TLR4.

### 3.6. AC Extract Treatment Facilitated Autophagy in Ipsilateral Hemisphere

**Figure 6:**
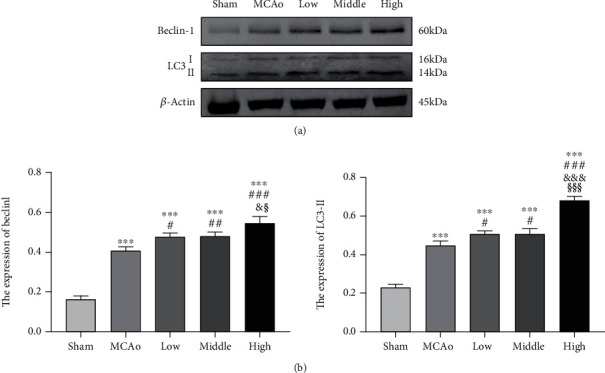
Upregulation of autophagy after ischemic stroke. (a) Representative western blotting belts for LC3-II and Beclin 1 and (b) quantitative analysis were presented. Data presented as mean ± standard deviation. ^∗∗∗^*p* < 0.001 vs. Sham; ^#^*p* < 0.05, ^##^*p* < 0.01, ^###^*p* < 0.001 vs. MCAo; ^&^*p* < 0.05, ^&&&^*p* < 0.001 vs. low; ^§^*p* < 0.05, ^§§§^*p* < 0.001 vs. middle.

Evidence showed that cytosolic form of LC3 (LC3-I) is conjugated to phosphatidylethanolamine (PE) to become an LC3-PE conjugate (LC3-II), which is required for the formation of autophagosomal membranes, and Beclin 1 is important for the localization of autophagic proteins to a preautophagosomal structure [13, 26]. As shown in Figure 6, we found expressions of LC3-II and Beclin 1 obviously elevated in MCAo group compared with sham group (*p* < 0.001), showing stroke triggered autophagy. Three dose groups further enhanced expressions of LC3-II and Beclin 1, especially high dose group was the most significant (*p* < 0.05, vs. low-middle dose group), which indicated that AC extract treatment enhanced autophagy in a dose-dependent manner poststroke.

### 3.7. AC Extract Treatment Attenuated Apoptosis in Ipsilateral Hemisphere

**Figure 7:**
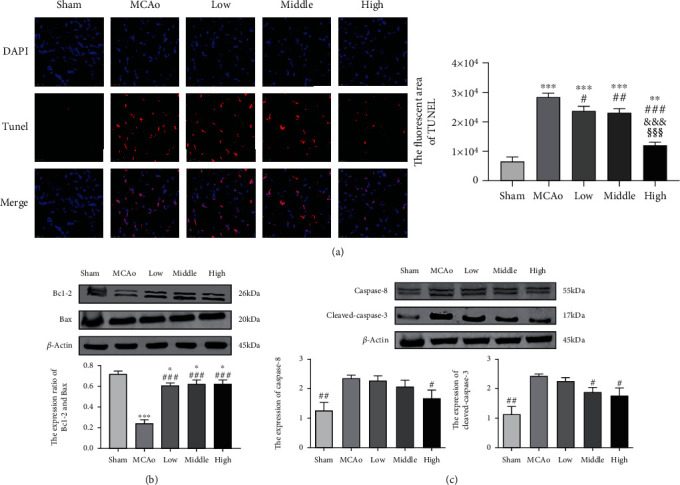
TUNEL staining and apoptotic pathway in ischemic penumbra. (a) Immunofluorescence staining with TUNEL (red) was presented (magnification, ×400) and quantitative analysis of TUNEL fluorescence. (b and c) Representative western blot belts for Bcl-2, Bax, Caspase-8, and cleaved-Caspase-3 and quantitative analysis. Data presented as mean ± standard deviation. ^∗^*p* < 0.05, ^∗∗^*p* < 0.01, ^∗∗∗^*p* < 0.001 vs. Sham; ^#^*p* < 0.05, ^##^*p* < 0.01, ^###^*p* < 0.001 vs. MCAo; ^&&&^*p* < 0.001 vs. low; ^§§§^*p* < 0.001 vs. middle. Scale bar 25 *μ*m.

As shown in Figure 7(a), cells with red TUNEL signal distributed in ischemic boundary zone, and quantitative analysis indicated that TUNEL expression was markedly ascended in MCAo group and yet three doses of AC extract groups dose-dependently attenuated TUNEL signals (*p* < 0.05). Moreover, western blotting results in Figures 6(b) and 6(c) indicated Bcl-2/Bax ratio gradually rose from low to high dose of AC extract groups compared with MCAo group (*p* < 0.001), and expressions of Caspase 8 and cleaved Caspase 3 in high dose group were lower than those in MCAo group (*p* < 0.05), which contributed to attenuation of apoptotic pathway.

## 4. Discussion

**Figure 8:**
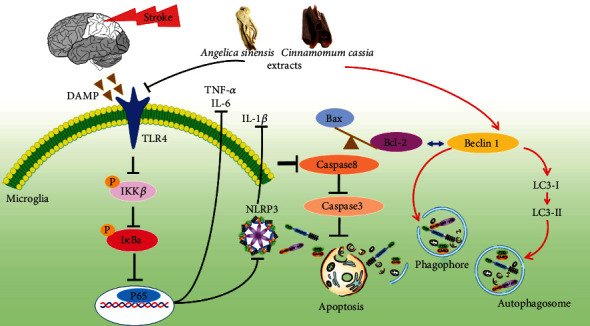
The schematic diagram of mechanisms of AC extract against neural injury by regulation of inflammatory and autophagic pathways after ischemic stroke.

Evidence suggests that inflammation and autophagy are closely associated with cerebral ischemic injury [27]. Our study firstly demonstrates that combination treatment of *Angelica sinensis* and *Cinnamomum cassia* is able to suppress neuroinflammatory response via inhibiting TLR4/NF-*κ*B pathway and NLRP3 inflammasome assembly and advance autophagy by elevating LC3-II and Beclin 1 expressions, as well as attenuate apoptosis by increasing Bcl-2/Bax ratio and decreasing caspase 8 and cleaved caspase-3 expressions, which is beneficial to exerting neuroprotective effect flowing cerebral ischemia (Figure 8).

Due to CBF interruption after stroke, several DAMPs (e.g., high mobility group box 1, heat-shock proteins, and nucleic acids) are derived from injured and necrotic cells release, and simultaneously, BBB disruption is implicated in leukocyte infiltration into the brain parenchyma, both of which contribute to the initiation of postischemic inflammation [6, 25]. Following ischemic progression, microglia can switch from an early anti-inflammatory phenotype to a proinflammatory phenotype, especially LPS and inflammatory cytokines can facilitate microglial transformation into proinflammatory phenotype [28]. In the current study, AC extract treatment can notably decrease Iba1 positive signal and increase CD206 positive signal, suggesting its inhibited proinflammatory efficacy on microglial cells after ischemic stroke.

On the membrane of microglia, there exist TLRs activated not only by DAMPs but also by pathogen-associated molecular patterns, e.g., LPS [29]. It is reported that TLR2 and TLR4 involve in inflammatory injury and subsequent ischemic damage poststroke, and TLR4-deficient mice present distinctly decreased infarcted volume, positive microglial number, and NF-*κ*B's p65 subunit after focal ischemic stroke [25, 29]. In the present study, we demonstrate that AC extract treatment notably reduces TLR4 expression whatever in ipsilateral hemisphere or in LPS-stimulated BV2 microglial cells by western blotting, and its efficacy on inhibition of TLR4 is similar to TLR4 siRNA's in vitro, showing AC extract can antagonize TLR4 activation.

The activated NF-*κ*B transcriptional pathway not only plays a predominant role in inflammatory aspects but also is critical to the regulation of apoptosis [30]. TLRs, NLRs, proinflammatory cytokines, and chemokine family, and so on, as “molecular switch” function of NF-*κ*B, phosphorylate I*κ*B*α* by the I*κ*B kinase (IKK) complex is composed of IKK*α*, IKK*β*, and regulatory IKK*γ*. Moreover, in IKK complex, it is reported that IKK*β* has higher capacities for IKK activation and NF-*κ*B induction than IKK*α* [31, 32]. Phosphorylation of I*κ*B*α* allows for NF-*κ*B nuclear translocation and DNA binding. Amount of literature show that suppression of TLR4/NF-*κ*B pathway attenuates brain edema and infarction volume and enhances antiapoptotic ability in MCAo model of rats [33]. In the present study, it suggests that treatment with AC extract obviously reduces phosphorylated IKK*β* and I*κ*B*α*, as well as nuclear P65 expression in a dose-dependent manner, contributing to inhibition of nuclear transcriptional activity of NF-*κ*B. Interestingly, in vitro, the number of LPS-stimulated BV2 microglial cells with nuclear P65 positive signal was not further altered after combining TLR4 siRNA with high dose group's CSF, suggesting AC extract mainly targets TLR4 instead of other targets in NF-*κ*B pathway.

TLRs and NLRs exert stepwise effects on the production of IL-1*β*, which suggests that TLR signaling yields pro-IL-1*β* and subsequently caspase-1 activated by NLRPs inflammasome cleaves it into IL-1*β* [6]. Recent reports evidence that NLRP3 inflammasome plays important mediating roles in inflammation and innate immunity response in central nervous system diseases [34]. In animal models of stroke, high NLRP3-inflammasome assembly and IL-1*β* expression occur, while NLRP3 deficiency improves neurovascular damage [35]. In the present study, it shows that treatment with AC extract downregulates expressions of NLRP3, ASC, and caspase-1 in a dose-dependent manner poststroke, which contributes to directly reducing the level of IL-1*β*, as well as other proinflammatory cytokine concentrations.

The study has indicated that TNF-*α*, IL-1*β*, and IL-6 secreted by activated microglia involve in neuronal cell apoptosis at stroke onset [36]. Hotter and colleagues [37] found IL-6 was an inflammatory marker of cerebral parenchymal damage. By ELISA assay, we discover there exists an interesting phenomenon on TNF-*α*, IL-1*β*, and IL-6 concentrations between in peripheral blood and brain parenchyma. In CSF and supernatant of ipsilateral hemispheric homogenizer, concentration of these inflammatory cytokines is obviously higher than that in serum. It reported that inflammasome proteins quickly diffuse into CSF after traumatic brain injury, so the quantity of inflammasome proteins in CSF may be used as biomarkers to predict long-term outcome or guide therapeutic interventions with the goal of lowering inflammasome protein concentrations [6]. Similar to the result, we hypothesize that alterations of TNF-*α*, IL-1*β*, and IL-6 concentrations in CSF are more suitable to represent inflammatory response after ischemic stroke. In the current study, AC extract treatment notably decreases TNF-*α*, IL-1*β*, and IL-6 concentrations in CSF, ipsilateral hemisphere, and peripheral blood, suggesting the ability of attenuating inflammatory cytokines. Moreover, in vitro, we found that treatment of high dose group's CSF plus TLR4 siRNA reduced TNF-*α*, IL-1*β*, and IL-6 concentrations in LPS-stimulated BV2, while it failed to further aggravate the attenuation compared with therapy alone. It once again demonstrates that AC extract treatment acts on inhibition of TLR4 followed by deactivating its downstream pathways.

Evidence indicates that upregulations of LC3-II and Beclin-1 expressions, as activated autophagy-related signals, play important roles in the formation of autophagosomes [13]. Our western blot results indicated that LC3-II and Beclin-1 expressions significantly elevated after AC extract treatment, which should contribute to the initiation of autophagy. The study discovers Bcl-2 binding to Beclin-1 after the onset of apoptosis inhibits autophagy, while activated autophagy conduces to injured neuronal cells to survive against apoptosis in rat MCAo model [13, 38]. Therefore, we hypothesize that upregulation of autophagy by AC extract treatment might contribute to appropriate degradation and digestion of integral cellular components, as well as isolation between Bcl-2 and Beclin-1. As a primary apoptotic pathway, Bax binding competition with Bcl-2 and other members of the Bcl-2 family in mitochondria membrane triggers activation of mitochondrial pathway following focal ischemia, which also directly releases apoptosis-inducing factor into nucleus resulting in chromatin condensation and large scale DNA fragmentation [39]. Additionally, after cerebral ischemia, Fas-associated death domain protein binding to procaspase-8 triggers activation of caspase 8; subsequently, caspase-3 is activated and causes caspase 3-dependent cell death [40]. Our result show AC extract treatment obviously increases the Bcl-2/Bax ratio and decreases caspase8/3 expressions, which contributes to antiapoptosis.

Caspase-1 activity, IL-1*β*, TNF-*α*, and NLRP3 have been demonstrated to be associated with modulation of apoptosis after stroke [9, 41, 42], causing activation of caspase-dependent pathways of cell death. In MCAo/reperfusion model, activated autophagic pathway attenuates NLRP3 inflammasome might be related to phagocytic actions and transportation into lysosomes [15, 16, 43], and TLR4-modulated NF-*κ*B pathway has a negative effect on autophagy regulation [27]. In the present study, elevated autophagy and suppressed TLR4-modulated NF-*κ*B pathway by AC extract treatment contribute to decreasing caspase-1, IL-1*β*, TNF-*α*, and NLRP3 expressions, which might be beneficial to attenuation of caspase-dependent pathways of apoptosis and explanation of relationship among inflammation, autophagy, and apoptosis, embodying multiple therapeutic advantages of AC extract on ischemic stroke. Of course, precise mechanisms need to be explored in the next study. Whatever these therapeutic effects ameliorate neurological functional outcomes after ischemia. Literature suggest that neurons in the infarct area can release damage signal to distal brain regions (e.g., thalamus) to trigger microglia-related secondary neurodegeneration [44]. Disruption of thalamic circuitry severely affects motor recovery [45]. Our results indicated that AC extract treatment significantly enhanced glucose utilization in thalamic lesion region, as well as elevated anti-inflammatory microglial phenotype, autophagy, and antiapoptosis, which contributes to the improvement of motor recovery due to integrity of thalamic circuitry poststroke.

Additionally, accumulating literature demonstrate active compounds extracted from *Angelica sinensis* and *Cinnamomum cassia* exert significant neuroprotective action by anti-inflammation or autophagy poststroke. Trans-cinnamaldehyde decreases iNOS, COX-2 expression, and NF-*κ*B signaling pathway against neuroinflammation in ischemia/reperfusion model [46], and cinnamaldehyde inhibits expressions of TLR4, TNF-receptor-associated factor 6, and nuclear translocation of NF-*κ*B in permanent MCAo model [47]. *Angelica sinensis* and its compounds have significant effects of anti-inflammation and antioxidative stress [48], and as a main active compound of *Angelica sinensis*, ferulic acid pretreatment exerted neuroprotective effects against apoptosis through activating 70 kDa heat shock protein (HSP70)/Bcl-2- and HSP70/autophagy-induced signaling pathways after focal cerebral ischemia [49]. In our study, we hypothesize these compounds from *Angelica sinensis* and *Cinnamomum cassia* play the main pharmacological role in neuroprotection. Precise pharmacodynamic research needs to be investigated in further study.

## 5. Conclusions

In summary, our study demonstrates that the extracts of *Angelica sinensis* and *Cinnamomum cassia* from oriental medicinal foods have significant capacities of inhibiting neuroinflammation, advancing autophagy, and antiapoptosis, which contributes to the improvement of neurological functional outcome. The mechanisms are associated with suppressing TLR4/NF-*κ*B pathway and NLRP3 inflammasome and enhancing LC3-II and Beclin-1 expressions after ischemic stroke. Therefore, we think AC extract is a novel neuroprotective agent of regulation of inflammatory and autophagic pathways for stroke treatment.

## Data Availability

Due to patent application and commercialization, the data used to support the findings of this study are available from the corresponding author upon request after publication of this article.
